# The effects of the exopolysaccharide and growth rate on the morphogenesis of the terrestrial filamentous cyanobacterium *Nostoc flagelliforme*

**DOI:** 10.1242/bio.026955

**Published:** 2017-09-15

**Authors:** Lijuan Cui, Haiyan Xu, Zhaoxia Zhu, Xiang Gao

**Affiliations:** School of Life Sciences, Hubei Key Laboratory of Genetic Regulation and Integrative Biology, Central China Normal University, Wuhan 430079, P. R. China

**Keywords:** Cyanobacteria, Morphology, Cultivation, Exopolysaccharide, Slow growth, Ecological improvement

## Abstract

The terrestrial cyanobacterium *Nostoc flagelliforme*, which contributes to carbon and nitrogen supplies in arid and semi-arid regions, adopts a filamentous colony form. Owing to its herbal and dietary values, this species has been overexploited. Largely due to the lack of understanding on its morphogenesis, artificial cultivation has not been achieved. Additionally, it may serve as a useful model for recognizing the morphological adaptation of colonial cyanobacteria in terrestrial niches. However, it shows very slow growth in native habitats and is easily disintegrated under laboratory conditions. Thus, a novel experimental system is necessary to explore its morphogenetic mechanism. Liquid-cultured *N. flagelliforme* has been well developed for exopolysaccharide (EPS) production, in which microscopic colonies (micro-colonies) are generally formed. In this study, we sought to gain some insight into the morphogenesis of *N. flagelliforme* by examining the effects of two external factors, the EPS and environmental stress-related growth rate, on the morphological shaping of micro-colonies. Our findings indicate that the EPS matrix could act as a basal barrier, leading to the bending of trichomes during their elongation, while very slow growth is conducive to their straight elongation. These findings will guide future cultivation and application of this cyanobacterium for ecological improvement.

## Abstract (precis)

**Summary:** By examining the effects of the exopolysaccharide and environmental stress-related growth rate on *Nostoc flagelliforme*, this study gained insight into the morphogenesis of the colonial cyanobacterium.

## INTRODUCTION

Cyanobacteria are an ancient and morphologically diverse group of photosynthetic prokaryotes that live in a wide variety of habitats (Schirrmeister et al., 2011). They contribute to carbon and nitrogen supplies in nutrient-poor environments (Veluci et al., 2006; Novis et al., 2007). In terrestrial niches, two phylogenetically neighbouring species, *Nostoc flagelliforme* and *Nostoc commune*, produce a large amount of exopolysaccharide (EPS) that encases their trichomes (Scherer and Zhong, 1991; Gao, 1998; Wright et al., 2001; Arima et al., 2012). The EPS thus facilitates their colony formation. *N. flagelliforme* exhibits a predominantly filamentous (hair-like or cylindrical) colony shape, while *N. commune* shows a lamellate colony shape. The EPS matrix is also crucial for enabling their colonies to absorb and retain moisture for growth, and protects cells against various environmental stresses (Tamaru et al., 2005; Nwodo et al., 2012; Rossi and De Philippis, 2015). Largely thanks to morphological differences, *N. flagelliforme* seems to exhibit greater adaptability to xeric environments than *N. commune* (Scherer et al., 1984; Scherer and Zhong, 1991; Gao, 1998; Gao et al., 2012). *N. flagelliforme* is distributed in arid or semi-arid regions that are characterized by high evaporation rate, intense solar radiation, drastic temperature differences and nutrient-poor soil, whereas *N. commune* is a cosmopolitan species that is mainly distributed in semi-arid or temperate regions. Thus, *N. flagelliforme* may serve as a useful model for recognizing the morphological adaptation of colonial cyanobacteria in terrestrial extreme environments. Owing to its herbal and dietary values, *N. flagelliforme* was overexploited before 2000, leading to severe deterioration of the native environments (Gao, 1998). Thus, elucidation of its morphogenesis will potentially enable its exploitation for ecological improvement (Aboal et al., 2016).

In native habitats, *N. flagelliforme* elongates by only 6% per year (Gao, 1998). Its artificial cultivation has been attempted many times, with little success (Gao et al., 2014). *N. flagelliforme* easily disintegrates under laboratory cultivation (Scherer and Zhong, 1991). In field cultivation, frequent and periodic watering can promote its growth to a certain extent, but often leads to its morphological transformation (from cylindrical to strip-like) or disintegration (Gao et al., 2012). The relative proportion of strip-like *N. flagelliforme* is also found to increase on relatively humid slopes or shaded lands. Liquid-cultured *N. flagelliforme* has been developed and serves as a valuable source for producing EPS (Gao and Ye, 2003; Su et al., 2008; Yu et al., 2010; Han et al., 2014a). In a long-term induction (1–3 months) by low ultraviolet (UV)-B radiation and/or periodic desiccation, single trichomes from the liquid culture could develop into colonial filaments 1–3 cm long on solid media (Feng et al., 2012). These observations seem to imply that, in addition to the EPS, environmental condition-associated slow growth or a proper growth rate is also an important aspect for affecting the morphogenetic process of natural colonies. In this study, we examined the morphological influence of both external factors on the shaping of aquatic-living colonies in appropriate culture conditions, to gain further insight into the morphogenesis of *N. flagelliforme*.

## RESULTS

### Microscopic observation of natural and aquatic-living colonies of *N. flagelliforme*

**Fig. 1.**
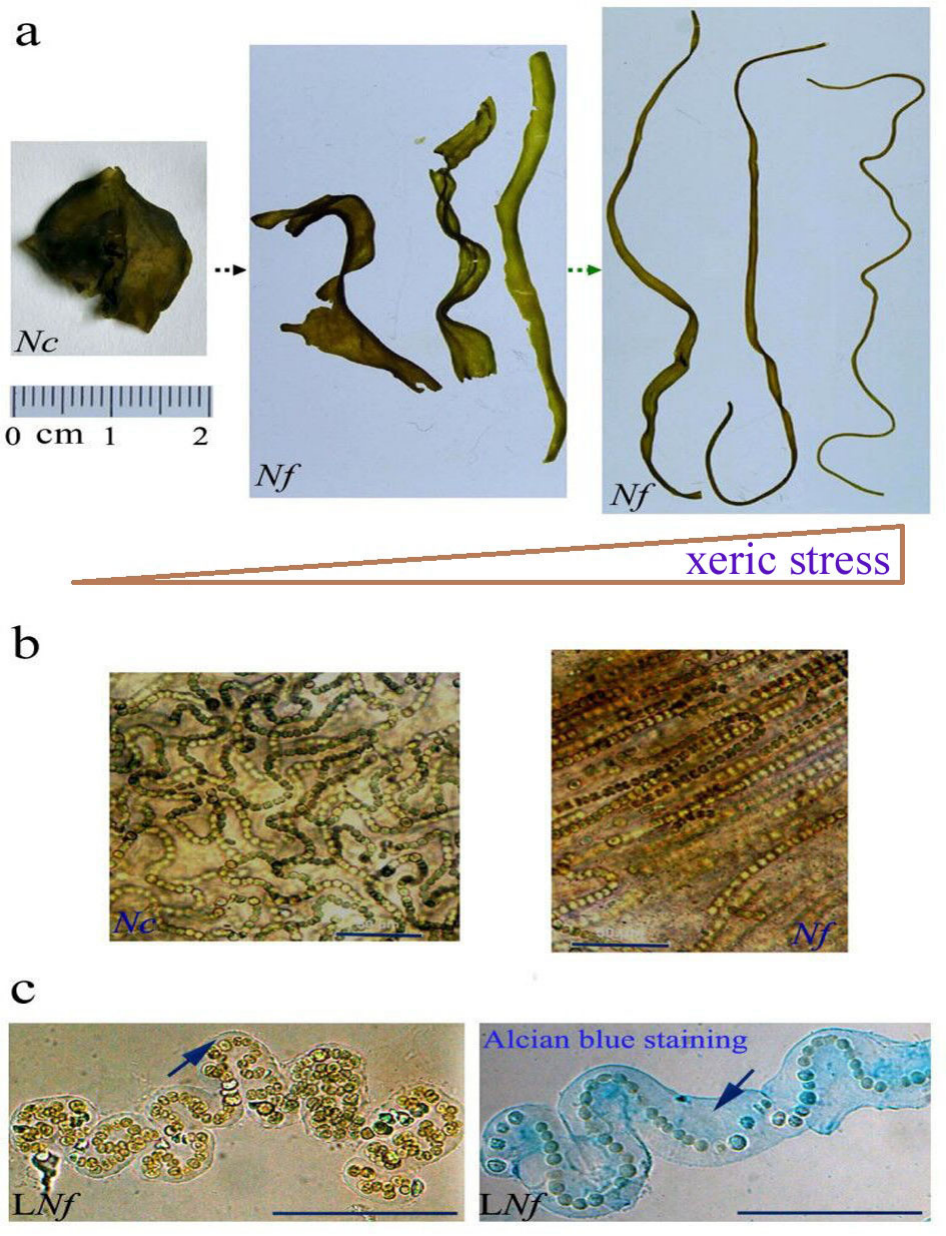
**Appearance and microstructure of *N. flagelliforme* and its close relative *N. commune*.** (A) Appearance of rehydrated natural colonies of both species. *Nc*, *N. commune*; *Nf*, *N. flagelliforme*. (B) Trichomes encased in the EPS matrix of natural colonies. (C) An individual micro-colony of liquid-cultured *N. flagelliforme.* Arrows indicate the capsular polysaccharide. L*Nf*, liquid-cultured *N. flagelliforme*. Scale bars: 50 μm.

Natural colonies of *N. flagelliforme* collected from various regions in western and west-northern China showed limited morphological diversity and were either cylindrical or strip-like when rehydrated (Fig. S1). In native habitats where *N. flagelliforme* and *N. commune* coexist, their junctional colonies were occasionally observed (Tang et al., 2003), implying an intermediate ecotype. Various molecular makers, including 16S rRNA, did not clearly separate the two species (Arima et al., 2012; Aboal et al., 2016). A potential morphological evolution between them, driven by the xeric environment, is shown in Fig. 1A. Under the microscope, the shape and arrangement of their trichomes were observed (Fig. 1B). The trichomes of *N. commune* were randomly twisted, which is apparently not conducive to the formation of an elongated or threadlike colony; in contrast, the trichomes of *N. flagelliforme* were generally arranged in parallel, which coincides with the colony form. This comparative difference in microstructure at least suggested a genetic variation in the directional control of cell division (Margolin, 2005). In liquid cultures of *N. flagelliforme*, microscopic aquatic-living colonies (or micro-colonies) could often be observed (Fig. 1C). A micro-colony consisted of a single trichome and its capsular EPS (CPS, which is tightly associated with the cell surface). Other secreted EPS was loosely dispersed in the solution, referred to as released EPS (RPS) (Pereira et al., 2009). The trichomes or micro-colonies also possessed an inherent feature of zig-zag (or Z-like) elongation, as shown in Fig. 1C. However, this Z-like bending growth showed large plasticity in liquid cultures, either severely bent or relatively straight, dependent on the culture phases or conditions, as implied by previous studies (Gao and Ye, 2003; Liu and Chen, 2003; Su et al., 2008; Gao et al., 2015) or shown in our experiments. Thus, a natural colony (filament) may be considered as an aggregation of many straight, elongated micro-colonies; and the effects of the EPS (especially CPS) and growth rate on the shaping of trichomes or micro-colonies would, to a certain extent, shed light on the morphogenesis of natural *N. flagelliforme*.

### Effects of the EPS on the shaping of trichomes or micro-colonies

**Fig. 2.**
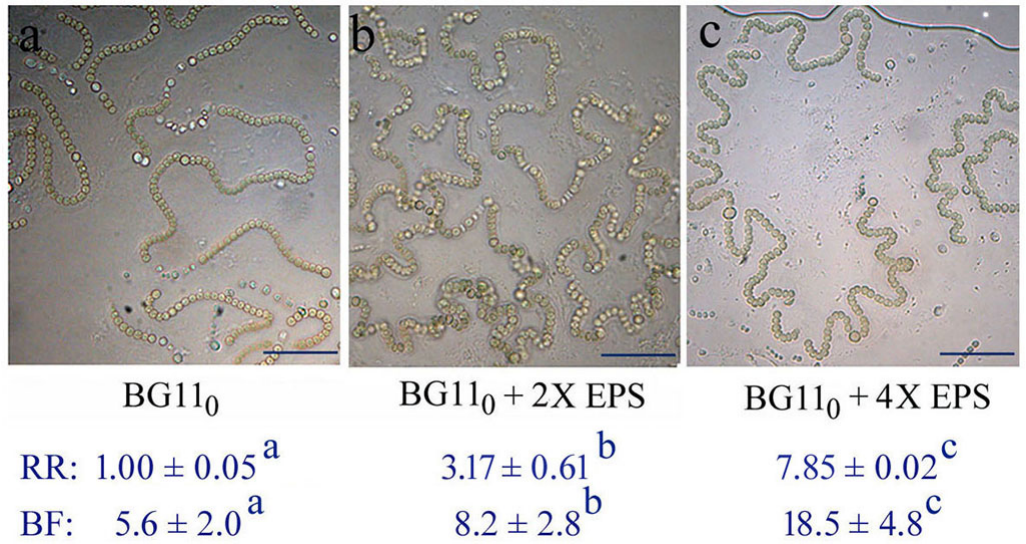
**Morphological changes of the *N. flagelliforme* trichomes after supplementation with additional EPS into the BG11_0_ cultures for a 12-day continuous cultivation.** 2X and 4X are two- and fourfold of the original total EPS concentration, respectively. RR, relative ratio of concentrations of CPS to Chl *a*; data are mean±s.d. (*n*=3). BF, bending frequency; data are mean±s.d. (*n*=40). Superscript letters (a,b,c) indicate significant differences (*P*<0.05, Tukey multiple comparison). Scale bars: 20 µm.

**Fig. 3.**
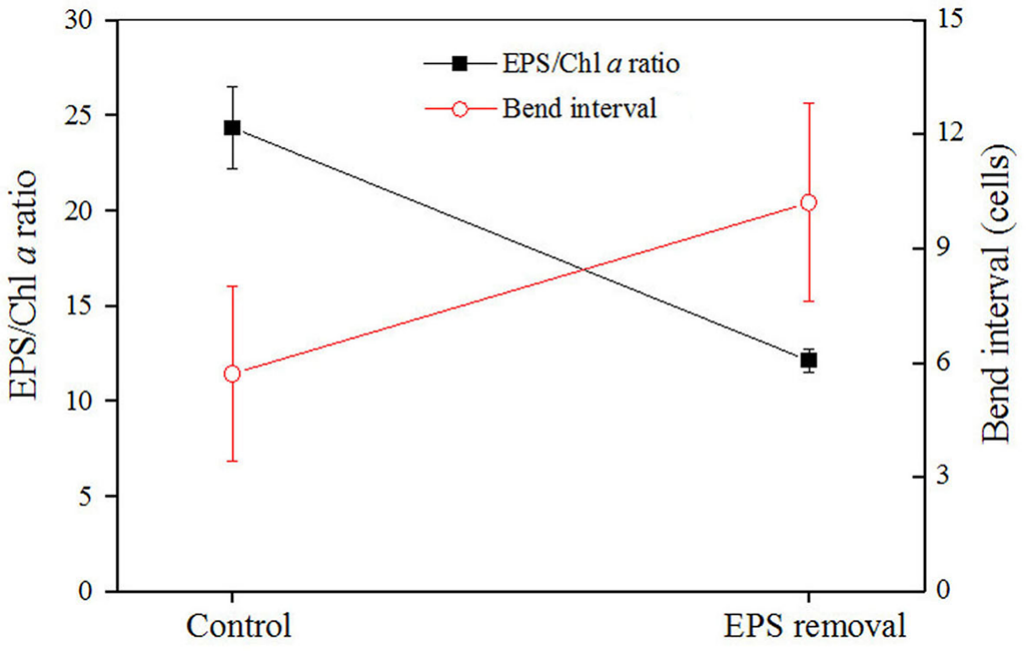
**Changes in the total EPS/Chl *a* ratio and bend interval of trichomes before and after the removal of polysaccharide from the BG11-cultured *N. flagelliforme* suspension.** Data are mean±s.d. (*n*=4, for EPS/Chl *a* ratio; *n*=40, for bend interval).

Many species of cyanobacteria, including *N. flagelliforme*, show a combined nitrogen-dependent rapid growth and EPS synthesis in liquid culture conditions (Otero and Vincenzini, 2003; Pereira et al., 2009; Yu et al., 2010). However, the trichomes or micro-colonies of *N. flagelliforme* could be induced to exhibit relatively straight elongation in nitrogen-free BG11_0_ solution (Rippka et al., 1979), as described in the Materials and Methods (Fig. 2A). The trichomes in BG11_0_ solution showed an approximately threefold lower growth than in nitrogen-rich BG11 solution (BG11_0_ supplied with 1.5 g/l NaNO_3_) under static cultivation. To evaluate the effects of CPS on the shaping of micro-colonies, we performed EPS addition (Fig. 2B,C) and removal experiments (Fig. 3). Following the supplementation of extra EPS (two- and fourfold of the original concentration, respectively) into the BG11_0_ cultures and then a period of adaptive cultivation, an enhanced bending growth of trichomes was obviously observed, as compared to the control (Fig. 2). The relative ratios of CPS to chlorophyll *a* (CPS/Chl *a* ratio) increased 3.2- and 7.9-fold, respectively. Correspondingly, the scored bending frequencies (bends per 100 cells) increased 1.5- and 3.3-fold, respectively. For most cyanobacterial strains, RPS and CPS contain similar sugar composition, and RPS originates from CPS (Ge et al., 2014). The changes in CPS/Chl *a* ratio in the present study also implied that the increased RPS concentration could lead to increased attachment of polysaccharide to cells. In the EPS removal experiment, some polysaccharides were rinsed from the BG11 cultures to evaluate the bending alteration of trichomes (Fig. 3). EPS removal (50% loss of the original EPS concentration) led to a 6.7-fold reduction in the CPS/Chl *a* ratio in the present study; meanwhile, a significant increase (1.8-fold) in the bend interval (average cell number per two adjacent bends) was observed, which corresponds to the reduction of bending frequency from 17.5 to 9.8. *N. flagelliforme* EPS possesses the physiochemical property of high intrinsic viscosity (Huang et al., 1998; Han et al., 2014b). Thus, these results may imply that the CPS acted as a direct barrier limiting the straight elongation of trichomes.

**Fig. 4.**
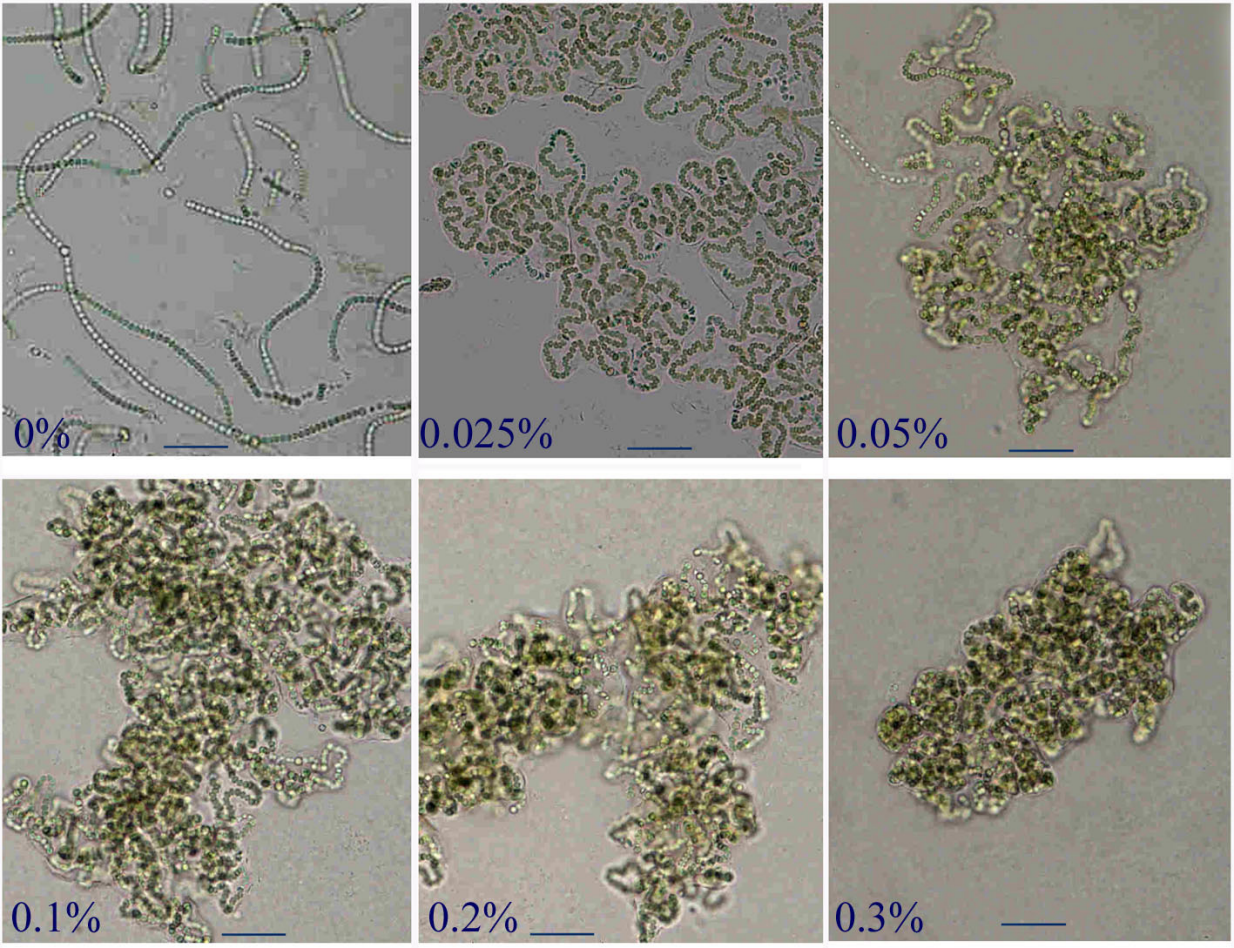
**Morphological changes of the *N. flagelliforme* trichomes cultured in BG11_0_ solutions gelated with various concentrations of agarose.** Scale bars: 50 μm.

To verify this potential physical effect on the shaping of trichomes, we further observed their morphological changes in the BG11_0_ media gelated with various concentrations of agarose (Fig. 4). The trichomes exhibited increased bending extents along with decreased fluidity of the media. In the 0.3% agarose medium, the trichomes were completely squeezed into an ellipsoidal lump. Taken together, these results implied that dense EPS could exert a physical barrier, causing the bending of trichomes during their elongation.

### Effects of environmental condition-related growth on the shaping of trichomes or micro-colonies

**Fig. 5.**
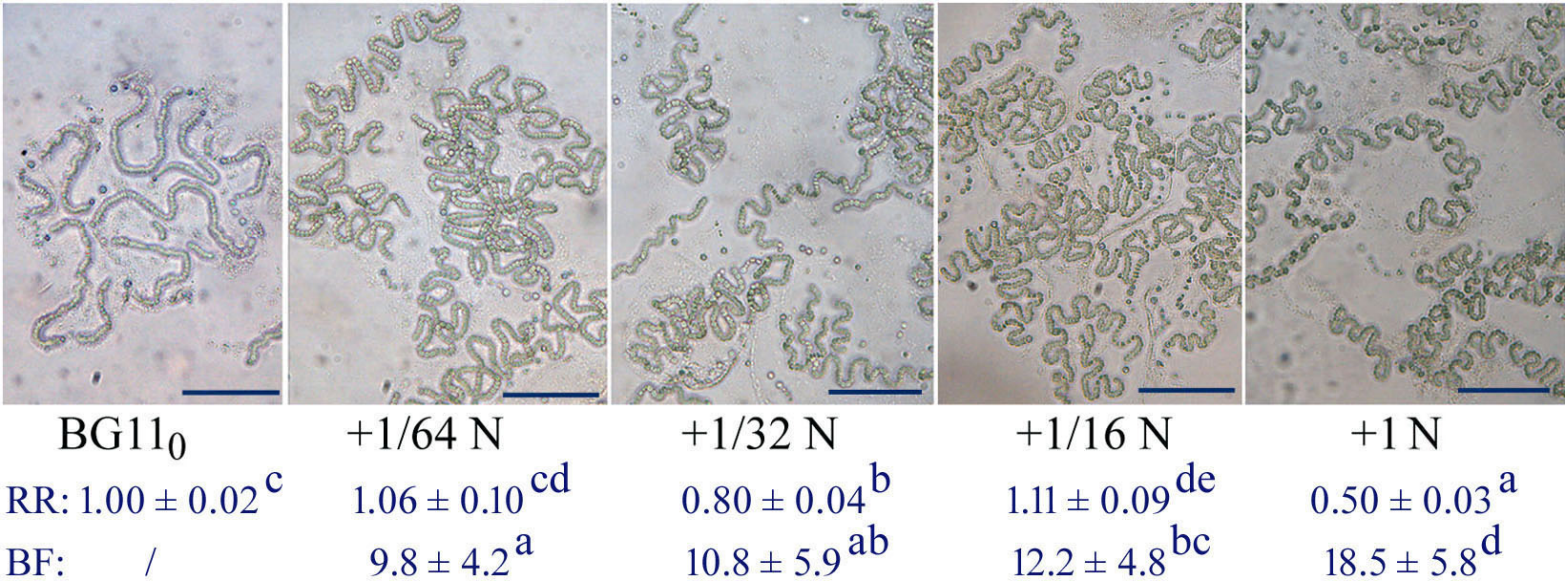
**Morphological changes of the *N. flagelliforme* trichomes in BG11_0_ solutions supplemented with various nitrogen levels relative to that in BG11 solution (1 N).** RR, relative ratio of concentrations of CPS to Chl *a*; data are mean±s.d. (*n*=3). BF, bending frequency; data are mean±s.d. (*n*=40). Superscript letters (a,b,c,d,e) indicate significant differences (*P*<0.05, Tukey multiple comparison). Scale bars: 50 μm.

The growth rate of aquatic-living *N. flagelliforme* is affected by factors including nitrogen availability, temperature, light wavelength and intensity (Yu et al., 2010; Han et al., 2014a). We evaluated the morphological effect of combined nitrogen-dependent rapid growth on *N. flagelliforme* trichomes by culturing an aquatic-living sample in BG11_0_ solutions supplemented with various concentrations of nitrogen source (Fig. 5). In these cultures, the CPS/Chl *a* ratios showed no obvious regularity; particularly, the relative ratio was lowest in the BG11 culture (onefold nitrogen level, 1 N level). However, the increased bending frequency was well correlated with the increased nitrogen level. It was also noteworthy that the Z-like elongation of trichomes was already quite obvious at a very low nitrogen level (1/64 N), implying a significant nitrogen-dependent incurrence of the trichome bending. Although nitrogen source can affect heterocyst metabolism and development (Muro-Pastor and Hess, 2012), the bends were rarely observed at the heterocyst position. The kinematic viscosities of hot water-extracted polysaccharides from nitrogen-rich and -free cultures of *N. flagelliforme* were also not much different (1.012 and 1.078 mm^2^ s^−1^, respectively), although their monosaccharide compositions were obviously different (Huang et al., 1998). Thus, the results at least implied that nitrogen source-related rapid growth was not conducive to the formation of straightly elongated trichomes.

**Fig. 6.**
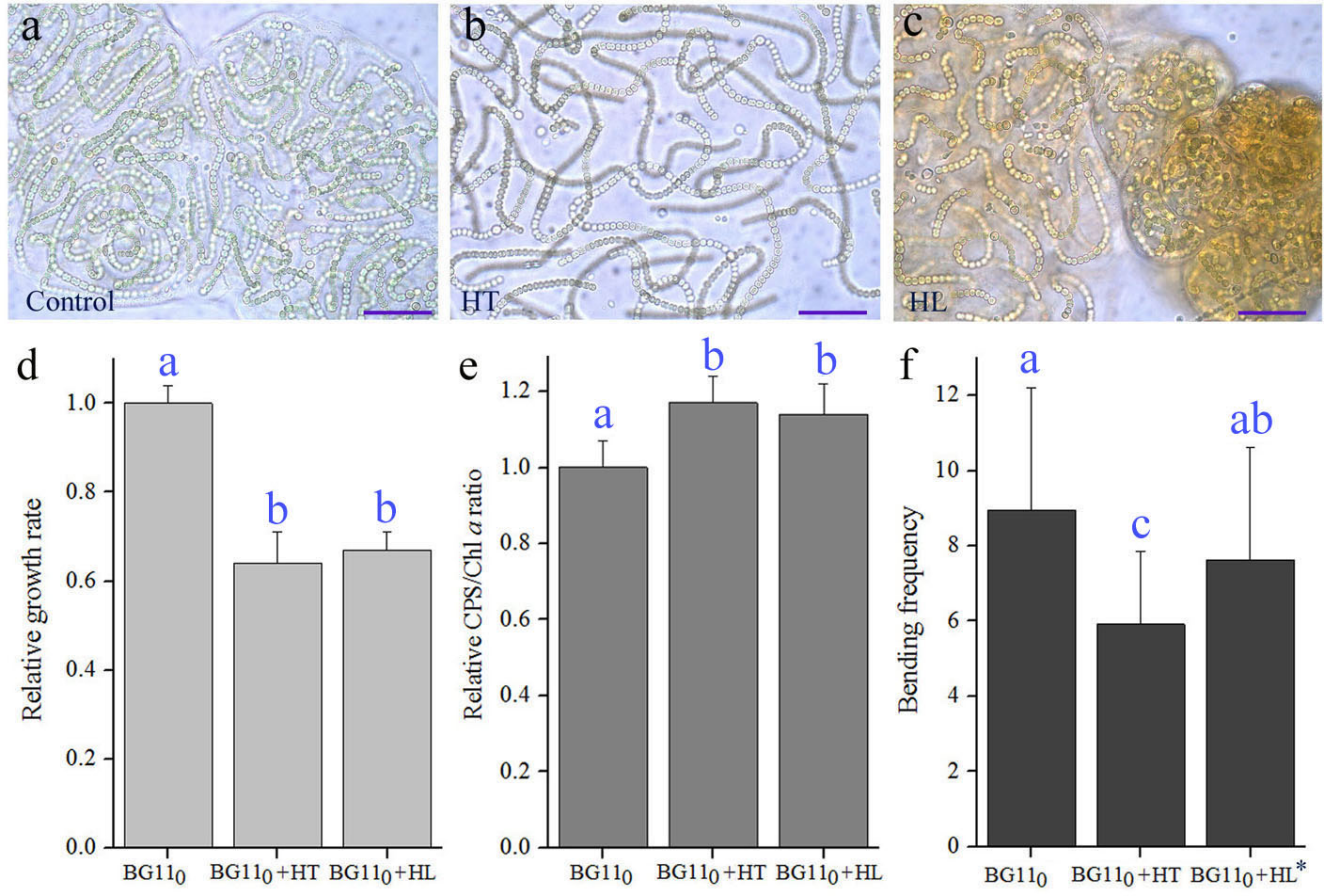
**Morphological changes of the *N. flagelliforme* trichomes upon high-temperature and high-light treatments.** (A) Control micro-colonies cultured at 25°C and 20 μmol photons m^−2^ s^−1^. (B) The micro-colonies cultured at 30°C and 20 μmol photons m^−2^ s^−1^. HT, high temperature. (C) The micro-colonies cultured at 25°C and 60-90 μmol photons m^−2^ s^−1^. HL, high light. (D) Relative changes in Chl *a* concentration. Data are mean±s.d. (*n*=3–4). (E) Relative changes in CPS/Chl *a* ratio. Data are mean±s.d. (*n*=3–4). (F) Bending frequencies of the trichomes. The asterisk indicates the loosely arranged trichomes under the HL condition. Data are mean±s.d. (*n*=40–50). Superscript letters (a,b,c) indicate significant differences (*P*<0.05, Tukey multiple comparison). Scale bars: 20 μm.

Generally, the optimal temperature and light intensity for the growth of liquid-cultured *N. flagelliforme* is 25°C and 40-60 μmol photons m^−2^ s^−1^ (Bi and Hu, 2004; Yu et al., 2010). The morphological effects of high temperature (30°C) and high light (60-90 μmol photons m^−2^ s^−1^) on the trichomes were evaluated, as compared to the control sample maintained at 25°C and 20 μmol photons m^−2^ s^−1^ (Fig. 6). After a 15 day culture, both unfavourable conditions led to a significant reduction in growth, but a significant increase in the CPS/Chl *a* ratio (Fig. 6D,E). The trichomes at 30°C appeared obviously looser and straighter than those at 25°C (Fig. 6B), consistent with the bending frequencies (Fig. 6F). However, high light seemed to exert more complex effects. The yellowish-brown colour of the trichomes (Fig. 6C) was suggestive of an increased carotenoid synthesis upon high light stress (Bi and Hu, 2004); some tightly arranged trichomes or globular micro-colonies existed (right, Fig. 6C). Nevertheless, those loose and straight trichomes were still observed in the high light condition (left, Fig. 6C). Their average bending frequency (7.6) showed a downward trend, relative to that of the control (9.0) (Fig. 6F); given that the control sample did not grow at the optimal light intensity and the treated sample had a higher CPS/Chl *a* ratio, this slight difference was still of significance. It was reported that stronger light (180 μmol photons m^−2^ s^−1^) did not allow the formation of colonial filament at 15, 25 or 30°C under aquatic conditions (Gao and Ye, 2003). Taken together, these results may also imply that environmental condition-related slow growth, in some instances, contributed to the straight elongation of trichomes.

## DISCUSSION

*N. flagelliforme* grows in arid or semi-arid regions, and its filamentous colony form should be a result of long-term adaptation to the xeric environment there. As an edible species, its artificial cultivation attracted high attention over the past few decades (Gao, 1998; Gao et al., 2014). A dilemma for its growth seems to lie in that scarce moisture limits its growth while substantial moisture becomes a burden (e.g. leading to its disintegration) (Gao et al., 2012). So far, there is no suggestive model to explain its morphogenesis. Liquid-cultured *N. flagelliforme* has been exploited for producing EPS (Gao and Ye, 2003; Su et al., 2008; Yu et al., 2010; Han et al., 2014a). In these studies, the relatively rapid growth and flexible morphology of micro-colonies have provided valuable basic information for us to consider their significance for recognizing the morphogenesis of natural colonies. In the present study, we gained clues that the EPS matrix could act as a basal barrier factor leading to the bending growth of the trichomes, while environmental stress-related slow growth was potentially conducive to their straight elongation. Rapid growth would at least enable the trichomes to encounter greater physical barrier, thus enhancing the bending frequency during their elongation. This may explain why the trichomes became severely bent and congested in the micro-colonies in favourable growth conditions, especially those with a supply of nitrogen (Gao and Ye, 2003; Su et al., 2008; Gao et al., 2015). In the late phase of either liquid or solid culture, the increased EPS may enhance the viscosity of the extracellular environments, thus also exacerbating the congestion of trichomes (Gao and Ye, 2003; Feng et al., 2012). A similar case was observed in *Nostoc sphaeroides* cultured in BG11_0_ solution (Deng et al., 2008). Although moderate mechanical agitation enhanced the total EPS production, the trichomes were observed to be long and straight, coinciding with the reduced CPS (Su et al., 2008). Therefore, a direct physical confinement of EPS on the elongating trichomes seems inevitable. In fact, the relatively straight micro-colonies used in this study were also induced in a slow growth condition (BG11_0_ solution and low light intensity) with a relatively low CPS/Chl *a* ratio. In addition, the Z-like elongation was also observed, as in other studies (Gao and Ye, 2003; Feng et al., 2012). Although this feature seems to be genetically weak, it should constitute the important basis for the formation of the filamentous colony, not the lamellate colony as in *N. commune* or the globular colony as in *N. sphaeroides* (Deng et al., 2008). In general, these experimental clues would improve our recognition of their potential influences on the morphogenesis of natural *N. flagelliforme*.

**Fig. 7.**
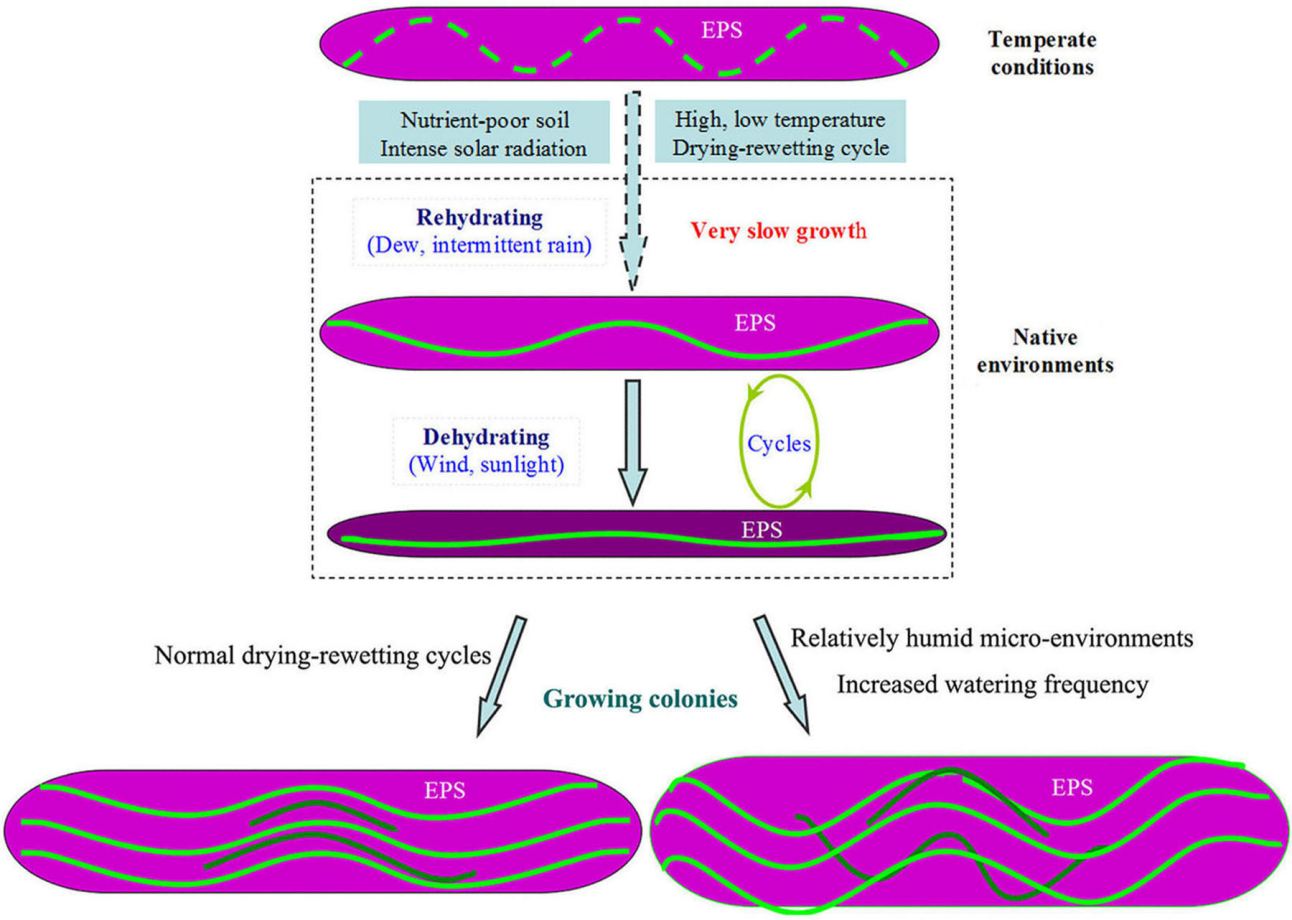
**A putative model for primarily describing the influence of external factors on the morphogenetic process of natural colonies of *N. flagelliforme.*** In the long-term evolution, trichomes (green lines) posses a weakly genetic Z-like elongation. The EPS acts as a basal barrier factor that potentially leads to the bending of the elongating trichomes, whereas environmental stress-related very low growth is conducive to their straight elongation. Periodic shrinking of the colony also contributes to the morphological re-modulation of trichomes. However, in relatively favourable environments, rapid growth leads to the increased bending of trichomes and the formation of a strip-like colony or even colony rupture.

In native environments, the specific morphology of *N. flagelliforme* should result from the combined effects of genetic and environmental factors. According to the above clues, coupled with previous observations (see Introduction), we propose a putative model for primarily describing the external or environmental influence on the morphogenetic process of natural *N. flagelliforme* (Fig. 7). Natural colonies showed a higher EPS/Chl *a* ratio of 50-90, higher than that in liquid cultures (Gao et al., 2015); also, the EPS extracted from natural colonies exhibited higher viscosity than that from laboratory suspension cultures (Huang et al., 1998). Thus, a stronger barrier for the elongation of trichomes may exist. In this circumstance, rapid growth is apparently not beneficial for their straight elongation. The unprecedented slow growth is recognized to be eco-physiologically crucial for the survival of colonial cyanobacteria in resource-poor and harsh environments (Sand-Jensen, 2014). Very slow growth, caused by the combined environmental stresses in nature (Gao, 1998), thus appears to play a dominant role in determining the shapes of trichomes and colonies of *N. flagelliforme.* In addition, periodic desiccation-rewetting processes not only slow trichome growth, but may also physically re-modulate the abnormally arranged trichomes in the growing colony, as implied by Feng et al. (2012). It is worth noting that this suggestive model may help to explain the previous failure of artificial cultivation of this edible cyanobacterium. To achieve rapid growth, favourable temperature and watering frequency are necessary; however, rapid growth will instead lead to the bending growth or protruding of trichomes and thus cause the colony rupture. Of course, the relatively favourable micro-environments may facilitate the formation of strip-like *N. flagelliforme* in native habitats.

The EPS matrix of natural colonies accumulates other sheath components, such as extracellular proteins (e.g. iron superoxide dismutase and acidic water stress protein) and UV-absorbing pigments (e.g. mycosporine-like amino acids and scytonemin) (Wright et al., 2005; Ferroni et al., 2010). In liquid cultures, both UV-absorbing pigments maintained at a very low level in the absence of UV irradiation (Yu and Liu, 2013) and at least acidic water stress protein could not be detected (W. Liu, L.C., H.X., Z.Z. and X.G., unpublished data). Thus, our model currently represents a preliminary and limited interpretation of the morphogenesis of *N. flagelliforme*. Why very slow growth benefits the straight elongation of the trichomes and colonies remains to be explored in the future. Here we put forward several possibilities: first, potentially alleviating the counter-force from the EPS, as mentioned above; second, a slow growth rate may allow this species to save enough energy to push the elongation of trichomes in a straight direction; third, it may facilitate the establishment of enough space with less physical barrier for the trichome elongation by secreted enzymes or via other means. Ultra-structural observation of natural *N. flagelliforme* showed that the cells were located in the voids of the EPS matrix (Liang et al., 2012), which potentially supports the third possibility. Epiphytic microorganisms can give rise to the disintegration of the EPS matrix under laboratory conditions (Gao and Ye, 2003; Gao et al., 2012); however, in native complex environments, their proper hydrolysis upon short-term rewetting may also facilitate the softening of the EPS matrix, thus benefiting the release of growth space. The molecular events with regard to the cell division control also need further investigation. Altogether, based on this current knowledge, mass cultivation of aquatic-living colonies and processing them with environmental resistance may be a relatively practical way to prepare enough ‘propagules’ for resource restoration and ecological improvement.

## MATERIALS AND METHODS

### Organisms and culture conditions

*Nostoc flagelliforme* (Berkeley and Curtis) Bornet and Flahault was collected in Sunitezuoqi, Inner Mongolia, China in 2012, and stored in dry conditions at room temperature. The air-dried sample was rehydrated in BG11 solution overnight for physiological recovery (Liu et al., 2010). Liquid-cultured *N. flagelliforme* was prepared by culturing the surface-disinfected sample in BG11 solution, as previously described (Feng et al., 2012). The liquid-cultured sample was inoculated in new BG11 medium in 250-ml conical flasks and cultivated statically at 25°C under continuous illumination of white fluorescent lights with a mean photon flux density of 20 μmol photons m^–2^ s^–1^ at the surface of the flasks. To prepare relatively straight trichomes or micro-colonies, the BG11 culture was collected by centrifugation (4000×***g***, 10 min) and washed with BG11_0_ solution three times. Then, the precipitated micro-colonies were subjected to static cultivation in 250-ml conical flasks at 22°C and the same light condition for 3–4 weeks. During the induction, the flasks were gently shaken two times per day. In the following experiments, samples were cultivated in 150-ml conical flasks for various treatments. The microstructures of natural colonies and micro-colonies were observed under a light microscope (Leica DM4000 B, Leica Microsystems, Wetzlar, Germany).

### EPS addition and removal experiments

The BG11_0_ cultures with induced relatively straight trichomes were used for the EPS addition experiment. Chl *a* was extracted with 95% ethanol overnight at 4°C in the dark and measured as previously described (Gao et al., 2014). Total EPS (RPS and CPS) was extracted with hot water and measured as previously described (Huang et al., 1998). After the *N. flagelliforme* suspension was centrifuged, RPS was extracted from the supernatant and purified in ethanol as described by Yu et al. (2010). CPS was extracted from the precipitated sample with hot water as previously described (Han et al., 2014a). The RPS was added to the BG11_0_ cultures, with two- and fourfold of the original EPS concentration, respectively, and after a 12 day culture, the final Chl *a* and CPS concentrations were determined. Also, the cell number between adjacent bends (<150 degree bend) was scored and calculated as the bending frequency (bend number per 100 cells). In this experiment, the final Chl *a* concentrations for the three cultures were ∼0.22 μg/ml and CPS concentrations were 0.43–3.52 μg/ml. Due to the variations in Chl *a* and EPS concentrations in each batch of experimental cultures, relative changes in the EPS/Chl *a* ratio were adopted for comparative analysis.

For the EPS removal experiment, 40 ml of the BG11 culture (0.28 μg/ml Chl *a*) was centrifuged (4000×***g***, 10 min) and the supernatant was removed. Then, the precipitated sample was cultured in 30 ml BG11_0_ solution. During the subsequent day, the sample was similarly precipitated and washed twice with 30 ml BG11_0_. On the third day, the Chl *a* concentration was 0.04 µg/ml. The initial and final EPS/Chl *a* ratios were calculated and the bend intervals of trichomes (cell number between adjacent bends) were scored. For better presentation, the bend interval was not calculated as the bending frequency in the figure in this experiment.

### Cultivation in agarose-gelated BG11_0_ solutions

BG11_0_ solutions of 50 ml were supplemented with various concentrations of regular agarose G-10 (1% gel strength, 750 g/cm^2^; Biowest, Madrid, Spain), ranging from 0 to 0.3%. After high-temperature sterilization and cooling (40-50°C), each solution was inoculated with 1 ml BG11_0_-cultured *N. flagelliforme* suspension. The cultures were subjected to static cultivation for 25 days. During the first week, the media were gently shaken for 1–2 h per day in a shaker (80 rpm/min) to properly disperse the trichomes. On the 25th day, the micro-colonies in each medium were observed under the microscope.

### Cultivation in nitrogen-supplemented solutions

BG11_0_ solutions were supplemented with various concentrations of combined nitrogen: 1/64, 1/32, 1/16 and onefold of the original NaNO_3_ concentration (1.5 g/l) in BG11 solution. Each 50 ml solution was further inoculated with 2 ml BG11_0_-cultured *N. flagelliforme* suspension. After 12 days of static cultivation, the trichomes were observed under the microscope and their bending frequencies were assayed. The final Chl *a* concentrations in the four cultures (0, 1/64, 1/32 and 1/16 N levels) were ∼0.10 µg/ml, and it was 0.32 µg/ml in the 1 N culture. The relative CPS/Chl *a* ratios were similarly calculated for comparison.

### Cultivation under high temperature and high light

The BG11_0_ cultures of 50 ml (0.01 µg/ml Chl *a*) were statically cultivated and subjected to high-temperature and high-light treatments for 15 days. Control samples were maintained at 25°C under continuous illumination of 20 μmol photons m^−2^ s^−1^, with a final Chl *a* concentration of 0.58 μg/ml on the 15th day. For the high-temperature treatment, the cultures were maintained at 30°C and in the same light condition. For the high-light treatment, the cultures were maintained at 25°C but exposed to continuous illumination of 60-90 μmol photons m^−2^ s^−1^. During the cultivation, the flasks were gently shaken two times per day. Correspondingly, relative growth rates, CPS/Chl *a* ratios and bending frequencies were determined, as compared to the control cultures.
